# Label-free quantitative proteomic analysis of serum exosomes from patients of renal anemia: The Good and the Bad of Roxadustat

**DOI:** 10.1186/s12014-022-09358-w

**Published:** 2022-06-11

**Authors:** Xiaoe You, Baochun Guo, Zhen Wang, Hualin Ma, Xinzhou Zhang

**Affiliations:** 1 The Second Clinical Medical College, Jinan University (Shenzhen People’s Hospital), Shenzhen, 518020 Guangdong China; 2 Department of Nephrology, Shenzhen Peoples Hospital The Second Clinical Medical College, The First Affiliated Hospital, Jinan University, Southern University of Science and Technology, Shenzhen, 518020 Guangdong China; 3 Shenzhen Key Laboratory of Kidney Diseases, Shenzhen People’s Hospital The Second Clinical Medical College, The First Affiliated Hospital, Jinan University, Southern University of Science and Technology, Shenzhen, 518020 Guangdong China

**Keywords:** Chronic kidney disease, Exosome, FG-4592, Proteomics, Roxadustat

## Abstract

**Background:**

Roxadustat is a new oral anti-renal anemia medication that works by stabilizing hypoxia-inducible factor (HIF) which can activate the expression of more than 100 genes in addition to genes related to anemia. However, the more potential molecular targets of roxadustat are not completely clear. Therefore, it is essential to further reveal its molecular targets to guide its clinical applications.

**Methods:**

We performed label-free quantification and LC-MS/MS to study the proteomic alterations in serum exosome of renal anemia patients before and after roxadustat therapy. Results were validated by PRM.

**Results:**

A total of 30 proteins were significantly changed after treatment with roxadustat. Among these proteins, 18 proteins were up-regulated (and 12 were down-regulated). The results are statistically significant (P < 0.05). Then, we validated the result by PRM, the results confirmed that TFRC, HSPA8, ITGB3, COL1A2, and YWHAZ were markedly upregulated, while ITIH2 and CFH were significantly downregulated upon treatment with roxadustat.

**Conclusions:**

TFRC and HSPA8 could be an important target of the action of roxadustat, and roxadustat may increase cardiovascular risk through its influence on platelet activation. Our results provide a theoretical basis for its wider clinical application and preventing expected side effects.

**Supplementary Information:**

The online version contains supplementary material available at 10.1186/s12014-022-09358-w.

## Background

Anemia is one of the frequent complications of chronic kidney disease (CKD), it is associated with poor outcomes and confers an increased mortality risk [1]. The limitations of existing drugs for renal anemia have raised our concerns and stimulated researchers' interest in finding alternative therapeutic approaches [2, 3]. In that case, Roxadustat (FG-4592), a new oral anti-renal anemia medication developed by FibroGen, was approved for marketing in China in December 2018 and China became the first country in the world to use it for the treatment of renal anemia.

Roxadustat is a hypoxia-inducible factor prolyl hydroxylase inhibitor (HIF-PHI) that regulates the expression of erythropoietin (EPO), hepcidin as well as others through HIF stabilization, thus promoting erythropoiesis at multiple levels [4, 5]. However, in addition to genes related to anemia, HIF can activate the expression of more than 100 genes, such as VEGF, HO-1, and TFRC which are related to a variety of biological and pathological processes [6–8]. It has been shown that HIF activation is intimately associated with the pathogenesis of many human diseases, such as pulmonary hypertension, myocardial ischemia, and cancer [9]. Therefore, we can make some educated guesses that with widespread clinical application roxadustat will disclose the therapeutic potential for more diseases and appear unobserved side effects via targeting other HIF downstream genes.

To confirm this conjecture, we decided to perform proteomic analysis of patients' exosomes to study the significantly differential protein before and after the roxadustat treatment. Exosomes are vesicles released by B cells, T cells, and other cells and exist in different body fluids. Proteins carried by exosomes can be transported between cells, participate in cell communication, material transport, and biochemical composition. Compared with traditional serologic markers, exosomes have greater sensitivity and are highly stable. This has become a research hotspot of precision [10]. LC-MS/MS is a technology of proteomics that has a better selectivity, higher sensitivity, and more accuracy than other methods, and the label-free quantitative method is considered to have higher proteome coverage capabilities [11].

**Fig. 1:**
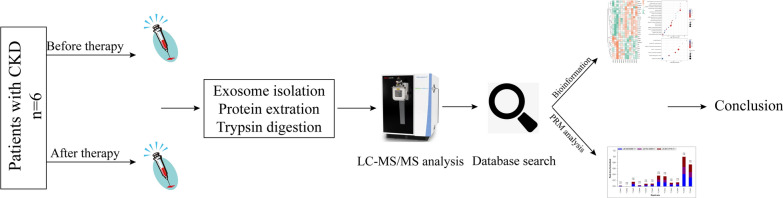
An overview of the experimental procedures used in this study

Therefore, we identified differentially expressed proteins (DEPs) of plasma exosomes from renal anemia patients before and after one month of roxadustat therapy by label-free quantitative LC–MS/MS proteomic analyses, and a variety of methods and bioinformatics tools were utilized to analyze the data in the proteome, including Gene Ontology (GO) analysis, Kyoto Encyclopedia of Genes and Genomes (KEGG) signaling pathway analysis, and protein–protein interaction (PPI) network analysis. An overview of the experimental proc edures used in this study is shown in Fig. 1. The DEPs identified may have the potential of assisting effective predictions of treatment responses in the future and provide an experimental basis for further revealing its molecular mechanism.

## Methods

### Patient assessments

Blood samples were collected from CKD patients who were hospitalized at Shenzhen People’s Hospital nephrology department from June 2021 to July 2021. The study was conducted by the declaration of Helsinki and the proposal has been approved by the Ethics Committee of the Second Clinical Medical College (Shenzhen People’s Hospital) of Jinan University. All subjects had signed the informed consent.

### Inclusion and exclusion criteria

The inclusion criteria were as follows: All the patients had a definitive diagnosis of CKD based on the General criteria of Kidney Disease Outcomes Quality Initiative (KDOQI), and Hemoglobin Levels Persistently below 11 g/dL [12].

The exclusion criteria were as follows: Patients with uncontrolled hypertension, active gastrointestinal bleeding, frequent blood transfusions in the past 6 months, and refractory iron deficiency anemia; Patients with malignancy; Patients with heart, liver, lung, and other important organ diseases; Patients with severe infection; Patients with drug allergy; Patients without regular medication intake.

### Collection of serum specimens

The samples (10 ml for each patient before and after medication, 6 patients in total) were centrifuged at 4℃ for approximately 10 min at 1000*g* (Centrifuge 5427 R, Eppendorf) and the supernatant was transferred to new tubes and stored at −80 °C.

### Exosome isolation

Serum samples were used for exosome isolation with exosome isolation kits (Umibio^®^, Cat. No: UR52136, China) according to the manufacturer's instructions. In brief, an initial spin was performed at 3000*g* 4 ℃ for 10 min and 10,000*g* 4℃ for 20 min for each sample (0.6 mL serum sample) to remove cellular debris and other impurities. The supernatant was diluted with pre-cooled 1× PBS (HyClone, SH30256.01, pH = 7.0), then 0.6 ml Blood PureExo Solution (BPS) was added. Mixtures were vortexed and incubated at 4℃ for up to two hours and then centrifuged at 10,000xg 4℃ for 60 min to precipitate exosome pellets. Pellets were resuspended with 1 ×PBS and purified with Exosome Purification Filter at 3000*g* 4℃for 10 min. The resuspension volume for exosome pellets was 6 μl for 0.6 ml starting volumes in this study. All exosomes were stored at −80℃ immediately after isolation until further analysis. Transmission electron microscopy (TEM) (JEOL, 1230) and nanoparticle tracking analysis (NTA) (Particle Metrix, ZetaView PMX 110) were performed to validate the exosome isolation and purification [13, 14].

### Protein extraction

Total 1.5 ml samples were removed from −80° C storage, and four volumes of lysis buffer (containing 8 mol/L urea (sigma, V900119) and 1% protease inhibitors (Merck, P8340)) were added to the ultrasonic processor instrument (PTM Bio, Hangzhou, China), after which the residual cell components were removed immediately by centrifugation (12,000×*g* at 4° C for 10 min). Then, the supernatant was transferred to a new tube, and the protein concentration was defined by a BCA kit (Beyotime, P0010) according to the manufacturer's instructions.

### Trypsin digestion

For digestion, 25 μL lysed protein solution (approximately 1 μg/μL) was reduced with 5 mM dithiothreitol (by adding 0.125 μL 1 M dithiothreitol (Sigma) for 30 min at 56 °C. Then the protein solution was alkylated with 11 mM Iodoacetamide (by adding 0.5 μL 0.55 M Iodoacetamide (Sigma)) for 15 min at room temperature in darkness. The protein sample was diluted by adding 100 μL 100 mM triethylammonium bicarbonate (TEAB) to a urea concentration less than 2 M. Finally, trypsin [(Promega)] was added at a 1:50 trypsin-to-protein mass ratio for the first digestion overnight and a 1:100 trypsin-to-protein mass ratio for a second 4 h-digestion. After the second 4 h enzymatic hydrolysis, the reaction was terminated by adding 10% trifluoroacetic acid (TFA) solution and adjusting the pH to 2–3. Then, the peptides were desalted by the C18 SPE column. Desalted peptides were frozen, drained off, and dissolved by mass spectrometry mobile phase A. Finally, 1 μg of peptides were taken to put on the machine.

### LC–MS/MS analysis

The digested 1 μg peptides were dissolved by liquid chromatography mobile phase A (containing 0.1% formic acid and 2% acetonitrile) and separated by EASY-nLC 1200 HPLC system [packed with 1.9 μm/120 Å ReproSil-PurC18 resins (Dr. Maisch GmbH, Ammerbuch, Germany)]. The mobile phase B was 0.1% formic acid in 90% acetonitrile. The liquid phase gradient was set as follows: 0–96 min, 4% ~ 20%B; 96–112 min, 20% ~ 32%B; 112–116 min, 32% ~ 80%B; 116–120 min, 80%B. The flow rate used was 500 nl/min. The peptides were injected into the NSI source for ionization and then analyzed using Exploris 480 mass spectrometer (Thermo). The voltage settings of the ion source were 2.3 kV, FAIMS compensation voltage was set to −45 V and −70 V. Orbitrap was used to detect and analyze the peptide parent ions and their secondary fragments. The first-order mass scans were performed with 60,000 resolutions, and the m/z range for the MS scans was 400–1200. The secondary mass spectrometry scan range had a fixed starting point of 110 m/z, and the secondary scan resolution was set to 30,000. TurboTMT was set to Off. The data acquisition mode was based on the data dependant scan (DDA) mode. To improve the effective utilization of the mass spectrum, the automatic gain control (AGC) was set at 75% and then the signal threshold was set to 10,000 ions/s and the maximum injection time was set to 100 ms with 30.0 s dynamic exclusion.

### Database search

Secondary mass spectral data were retrieved using PD2.4 (v2.4.1.15). Retrieval parameter settings: The database was Homo sapiens (75,777 sequences), the anti-library was added to calculate the false discovery rate (FDR); the digestion method was set to trypsin (Full); the number of missed cleavages was set to 2; The minimum peptide length was set to 6 amino acids and the maximum length was set to 3 amino acids; the tolerance values of the mass error for the primary precursor ion was set to 10 ppm, and the error tolerance of the mass of the second fragment ion was to 0.02 Da; Carbamidomethyl cysteine (C) was set as a fixed modification and Oxidation (M), Acetyl (N-terminus), Met-loss (M), Met-loss + acetyl (M) as a variable modification. and the FDR for the protein identification and peptide spectrum matches identification was adjusted to 1%.

### Bioinformatics methods

GO annotation of the proteome was derived from the UniProt-GOA database (http://www.ebi.ac.uk/GOA/). Proteins were classified by GO annotation based on three categories as follows: molecular function, biological process, and cellular component. The protein domain functional descriptions identified in this study were annotated by InterProScan, which analyzes the data through both the InterPro domain database (http://www.ebi.ac.uk/interpro/) and the protein sequence alignment. The pathway analysis was performed using the KEGG pathway (KEGG protein database: http://www.kegg.jp/kegg/pathway.html). The KEGG database was used in this study to identify enriched pathways and to test the enrichment of the different proteins against all identified proteins by a two-tailed Fisher’s exact test. The pathways with corrected P values < 0.05 were considered significant and were separated into individual categories. All differentially expressed protein accession numbers or sequences were examined thoroughly by the STRING database (version 10.5) for convenient presentation and protein–protein interaction analysis.

### PRM analysis

Preparations were conducted at the initiation of Parallel reaction monitoring (PRM) analysis including protein extraction and trypsin digestion described in previous procedures. The liquid phase gradient consisted of an increase from 6 to 23% solvent B in the first 38 min, 23% to 35% in the next 38 to 52 min and a rise to 80% from 52 to 56 min, all at a constant flow rate of 700 nL/min for 56 to 60 min on an EASY-nLC 1000 UPLC system. Afterward, the peptides were analyzed by MS/MS in Q Exactive^™^ Plus [(Thermo Fisher Scientific (USA))] combined and connected to the UPLC and then they went through the NSI source with the electrospray voltage preset at 2.0 kV. The range of the m/z scan was adjusted from 350 to 1000 for a full scan, and the resulting peptides were detected by Orbitrap at a resolution setting of 17,500. A data-independent mode was alternated between one MS scan and 20 scans. AGC was set at 3E6 mode for the whole MS, and 1E5 mode was specified for MS/MS. The maximum IT was set at 20 ms for full MS and auto for MS/MS. The isolation window was set to 1.6 m/z for MS/MS. The resulting MS data were processed using Skyline (v.3.6). Peptide settings: enzyme was set as Trypsin [KR/P], Max missed cleavage set as 2. The peptide length was set as 8–25, Variable modification was set as Carbamidomethyl on Cys and oxidation on Met, and max variable modifications were set as 3. Transition settings: precursor charges were set as 2, 3, ion charges were set as 1, 2, ion types were set as b, y, p. The productions were set as from ion 3 to last ion, the ion match tolerance was set as 0.02 Da.

### Statistical analysis

SPSS17.0 software was used for data processing and analysis. The measurement data were expressed as mean ± standard error of the mean (SEM). A Paired-sample t-test was used for characteristics of the samples between the two groups. P < 0.05 was considered statistically significant.

## Results

### Population

Six participants were involved in this study. The clinical characteristics of patients were listed in Table 1. As we can see, an upward trend in hemoglobin (HB) was observed after 1 month of roxadustat treatment (p = 0.05). In addition, the low-density lipoprotein level of patients was significantly lowered, and total cholesterol and triglycerides also showed a decreasing trend. It is well known that dyslipidemia is an established risk factor for cardiovascular disease in patients with CKD, the improvement of blood lipids may provide clinical benefit. These results were concordant with the findings of previous studies [15, 16].

**Table 1:** Demographic and clinical data of the six renal anemia patients in our study

	T0	T1	P-value
Age [years]	49.67 ± 18.82	49.67 ± 18.82	–
Sex (M/F)	1/5	1/5	–
Hemoglobin(g/l)	82.67 ± 12.04	90.67 ± 15.80	0.05
Erythrocytic count	2.83 ± 0.50	3.12 ± 0.87	0.20
HCT	26.90 ± 5.96	28.43 ± 6.49	0.38
Low-density lipoprotein(mmol/l)	2.12 ± 0.53	1.45 ± 0.57	0.02
Triglycerides(mmol/l)	2.44 ± 1.66	1.78 ± 1.6	0.10
Total cholesterol(mmol/l)	3.91 ± 0.56	2.66 ± 0.45	0.07

Table showed levels of HB, erythrocytic count, HCT, and lipids before (T0) and after 1 month (T1) of roxadustat treatment. The average (± standard deviation) values are reported

### Characterization of exosomes

**Fig. 2:**
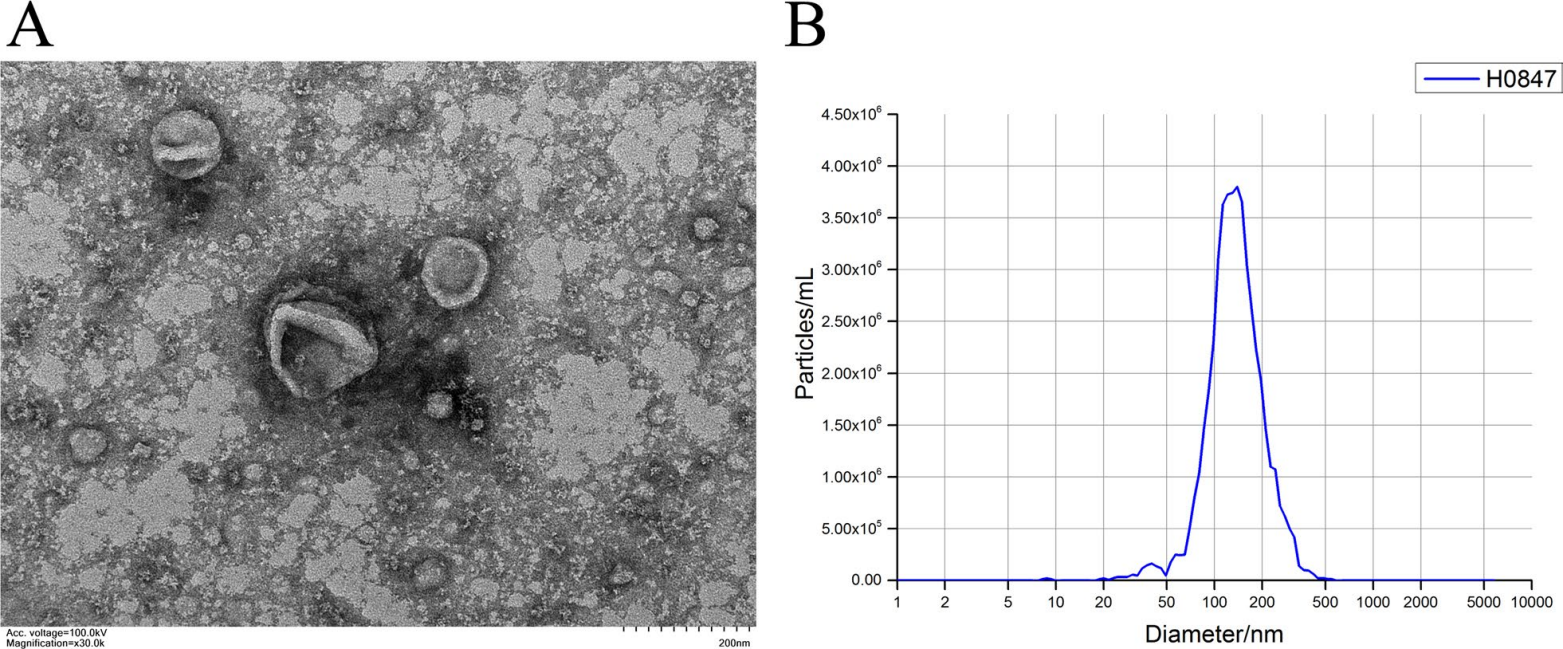
Isolation and purification of serum exosomes. **A** Representative transmission electron microscopy (TEM) images. TEM demonstrates that isolated samples consisted of vesicles with typical membrane morphology. **B** Representative nanoparticle tracking analysis (NTA) of isolated exosomes. NTA indicates that most vesicles had a size of 30–150 nm in diameter

TEM and NTA were performed to validate the exosome isolation and purification [17, 18]. Electron microscopy revealed a homogenous mixture of cup‐shaped, rounded nanovesicles with diameters varying between 30 and 150 nm (Fig. 2A**)**. Subsequently, NTA was carried out using ZetaView PMX 110 (Particle Metrix, Meerbusch, Germany) and corresponding software ZetaView 8.04.02, which confirmed the size distribution with a mean = 144.5 nm (Fig. 2B). The above two confirmed the successful isolation and purification of exosomes [19].

### Proteome Profile in exosomes before and after roxadustat therapy

**Fig. 3:**
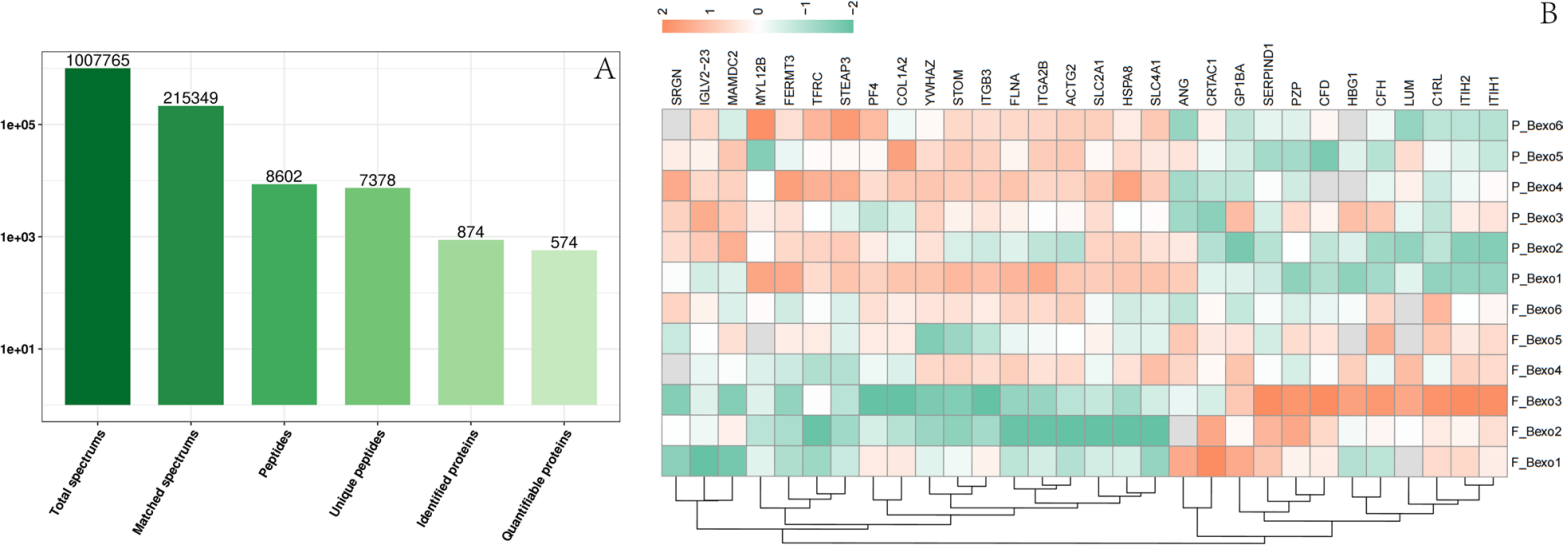
Protein identification results. **A** The total number of peptides and proteins identified. **B** Heat map of the DEPs in the CKD patients

Database search was performed using Proteome Discover software (v2.4.1.15) to calculate and match with the human database. Meanwhile, the anti-library was added to calculate the false discovery rate (FDR). Finally, 874 proteins were identified, from which 574 proteins were quantified **(**Fig. 3A). The detailed method of quantification is as follows: Screen the proteins with at least two unique peptides, normalize the Intensity, and calculate the ratio after calculating the relative quantitative values of the sample. To check the acquired MS data, we verified that the mass error was between—11 and 4 ppm, which meets the requirement of mass accuracy. Most peptides ranged from 7 to 20 amino acids in length, which is consistent with the basic principle of trypsin digestion. The cutoff for the identification of DEPs was a P-value < 0.05. A greater than 1.5-fold change was termed upregulation, while a fold change of < 1/1.5 was termed downregulation for DEPs (Fig. 3B). The results showed that 30 differential proteins were identified, 18 were up-regulated, and 12 proteins were down-regulated (see Additional file 1).

### Functional classification of subcellular localization and GO analysis of the DEPs

**Fig. 4:**
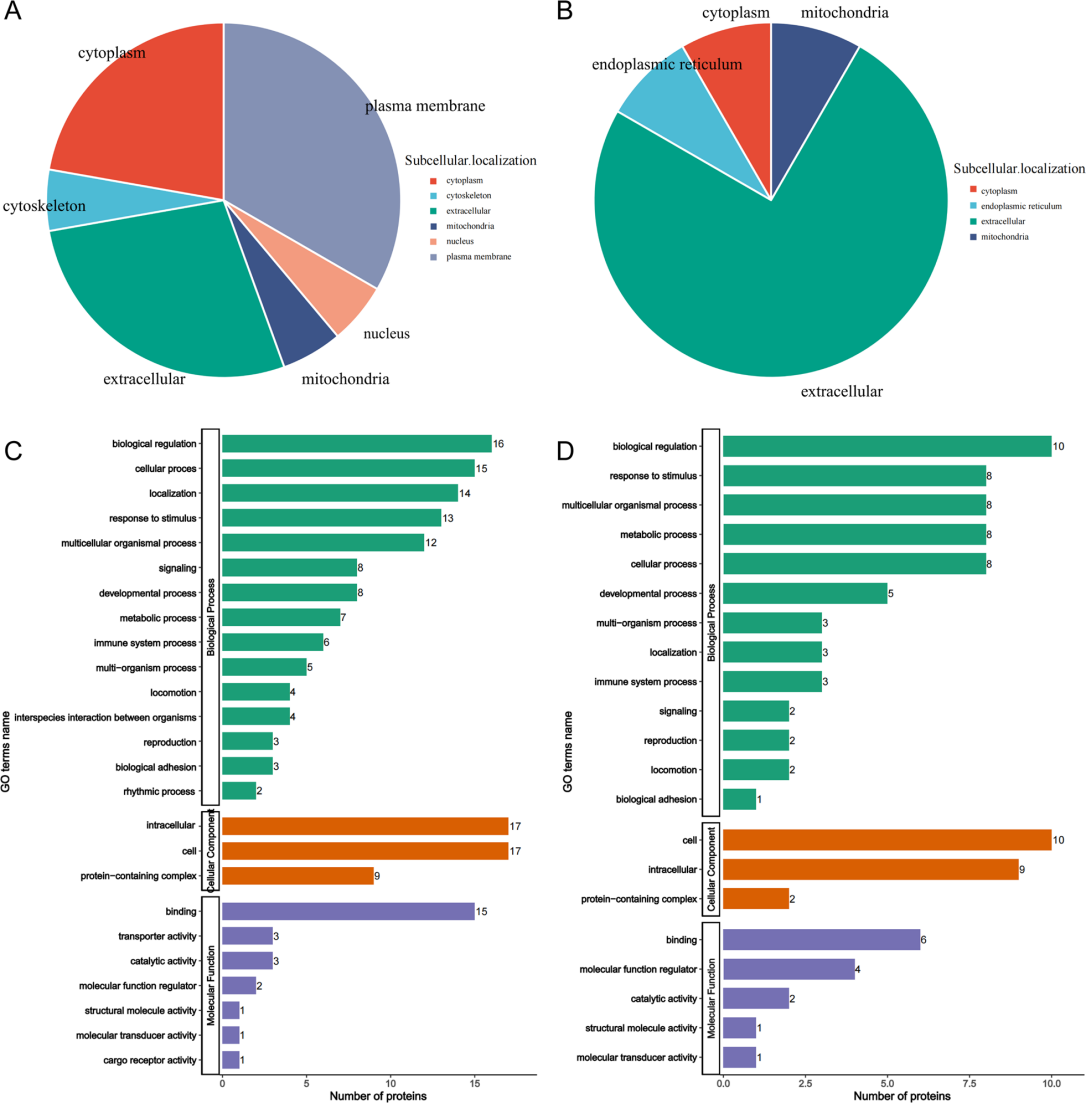
Subcellular localization and GO classifications in the exosome proteins. **A** Subcellular localization of up-regulated proteins. **B** Subcellular localization of down-regulated proteins of the DEPs. **C** GO classifications of up-regulated proteins. **D** GO classifications of down-regulated proteins

By analyzing the subcellular localization of DEPs, the upregulated proteins were found to be mainly distributed in the plasma membrane, cytoplasm, extracellular, cytoskeleton, mitochondria, and nucleus. And the majority of the down-regulated proteins were distributed in the extracellular, mitochondria, endoplasmic reticulum, and cytoplasm (Fig. 4A, B). We observed no meaningful differences between upregulated and downregulated proteins.

To evaluate the overall characteristics of DEPs, we performed GO functional classification for DEPs (Fig. 4C, D). In the molecular function category, DEPs were primarily related to binding, transporter activity, and catalytic activity. In the cellular component category, DEPs were mainly located in cell, intracellular and protein—containing complex. In the biological progress category, DEPs were mainly associated with biological regulation, cellular process, localization, response to stimulus, metabolic process. Notably, the immune system process category contained 9 DEPs (30%). Among them, multiple DEPs involved in the inflammatory response, including CRTAC1, ITIH1and ITIH2. Yinghong Ji et al. proved that the inhibition of CRTAC1 alleviated oxidative stress and inflammation response by western blot analyses [20], and ITI family members have been demonstrated to be both positive and negative acute-phase proteins that are activated in the inflammation setting [21]. These proteins were specifically down-regulated, suggesting a role of immunology and inflammation in renal anemia and CKD, and may illustrate that roxadustat has an anti-inflammatory effect.

### GO and KEGG analysis of the differential expression proteins

**Fig. 5:**
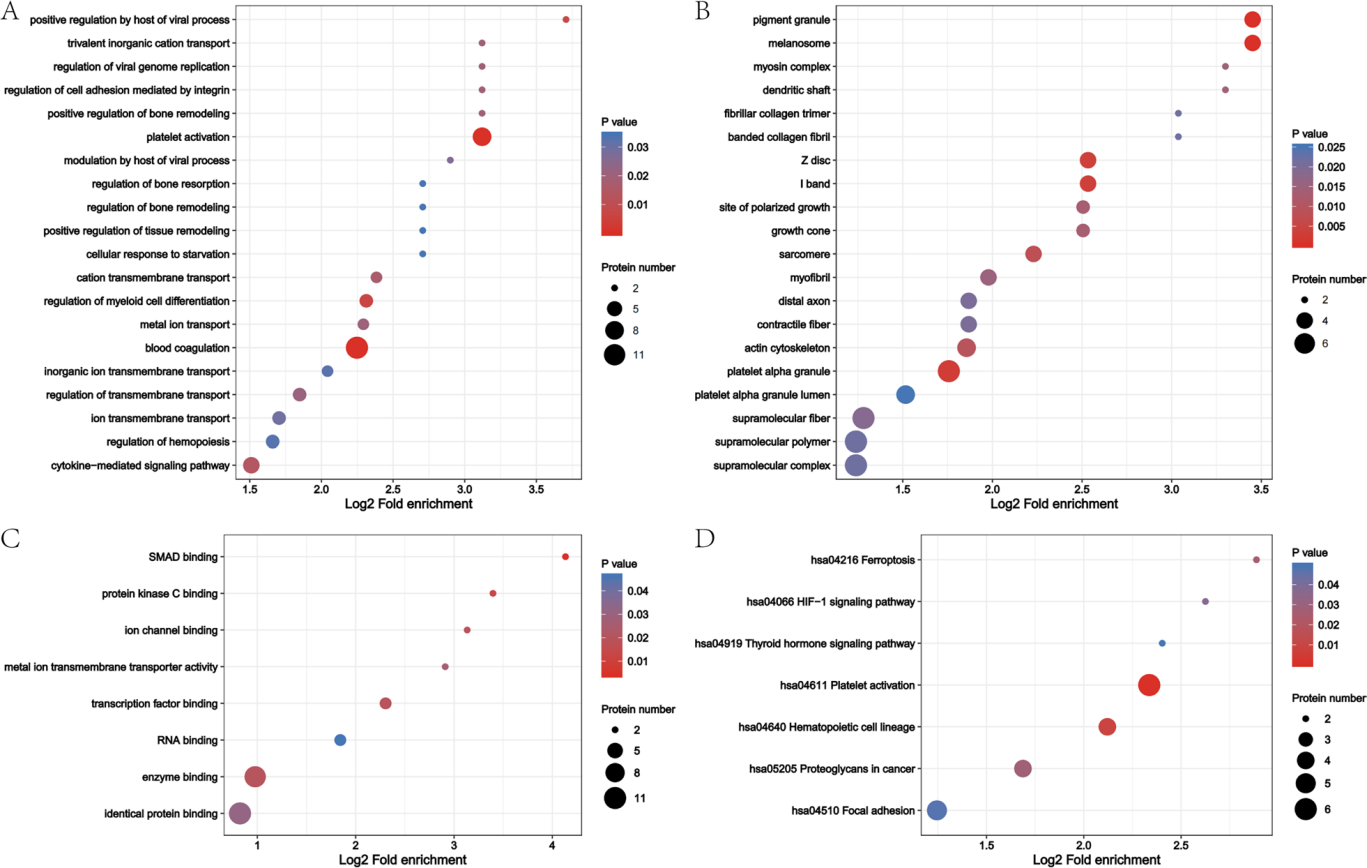
GO and KEGG analysis of the differential expression proteins. **A** Biological process of DEPs. **B** Cellular component of DEPs. **C** Molecular function of DEPs. **D** KEGG pathway

For an overview of the function of all detected DEPs between them, a GO enrichment analysis was performed for the DEPs detected. The DEPs were classified into the biological process (Fig. 5A), cellular component (Fig. 5B), and molecular function (Fig. 5C) categories. Most proteins were in the platelet alpha granule lumen, supramolecular polymer, and melanosome according to the classification of cell components classification. In addition, in the classification of molecular functions, most DEPs were involved in identical protein binding, enzyme binding, and transcription factor binding categories. Finally, the DEPs were associated with blood coagulation, platelet activation, metal ion transport, positive regulation by host of viral process, and regulation of hemopoiesis in terms of biological process. Our initial analyses found significant alterations in the proteome related to coagulation platelet activation, which might contribute to a hypercoagulable state in CKD patients. Further study is warranted to address whether roxadustat treatment of CKD patients affects clinical cardiovascular risk. At the moment of the SARS-CoV-2 pandemic, Roxadustat was proved that can reduce ACE2 expression and inhibit SARS-CoV-2 entry and replication in lung epithelial cells via a HIF-1α-dependent pathway [22], this may be part of the effect of roxadustat on the virus.

The KEGG-based functional enrichment analysis showed that the DEPs were significantly changed in 5 processes (Fig. 5D), including hsa04611 Platelet activation, hsa04640 Hematopoietic cell lineage, hsa04066 HIF-1 signaling pathway, hsa04216 Ferroptosis. The HIF-1 signaling pathway is the well-known pathway of Roxadustat, and other pathways may also have an important association with the molecular mechanism of roxadustat. There were other enriched KEGG functional pathways. For example, PI3K-Akt signaling pathway, Proteoglycans in cancer, and Focal adhesion and thyroid hormone signaling pathway. Indeed, Roxadustat was reported to lead to hypothyroidism, this is due to Roxadustat having structural similarity with T3 and is a selective activating ligand for thyroid hormone receptor-β possibly suppressing thyroid-stimulating hormone (TSH) release. But no hypothyroidism was detected in this study, the insufficient sample size may be one of the reasons for it.

### Protein–protein interaction (PPI) network of the DEPs

**Fig. 6:**
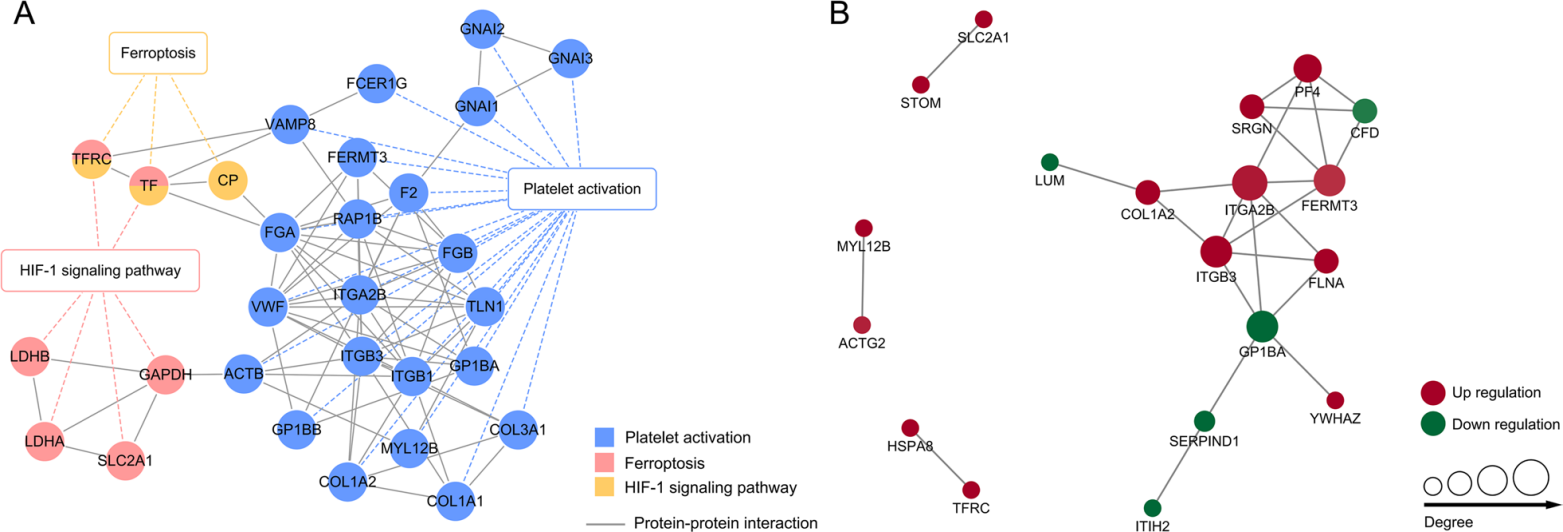
PPI network of the different signaling pathways **A** and the differentially expressed proteins **B**. The circles in the figure represent differentially expressed proteins, and different colors represent the differential expression of proteins (green is down-regulated protein, red is up-regulated protein). The darker the color, the greater the multiple of difference

Combined with the results of Roxadustat before, we select 3 signaling pathways that may be associated with CKD or renal anemia: Platelet activation (see Additional file 2), HIF-1 signaling pathway (see Additional file 3), and Ferroptosis (see Additional file 4). To further explore the interrelationship of the different pathways, we constructed a PPI regulatory network that contained proteins that have potential implications with the expansion of the sample size. As shown in Fig. 6A, it can be intuitively seen that the proteins were related to each other, TF and TFRC can simultaneously target two signaling pathways mediated by roxadustat. At the same time, the number of proteins involved in the platelet activation pathway is substantial, which verifies the above conjecture.

DEPs were compared by the STRING protein–protein interaction database, according to a confidence score > 0.7 (high confidence). Then these proteins were made into the PPI network diagram [23] (Fig. 6B) and further screened out 3 hub proteins with high connectivity degree, namely, ITGA2B, ITGB3, and FERMT3. Based on the previous studies, the ITGA2B gene encodes the aIIb subunit (GPIIb), whereas the ITGB3 gene encodes β3 (GPIIIa), and GPIIb/IIIa is the central receptor of platelet aggregation and plays a primary role in both platelet adhesion and thrombus formation at the vascular injury site. Thus, ITGA2B and ITGB3 play an important role in normal platelet function [24]. Similarly, FERMT3 also referred to as kindlin-3, is considered essential for integrin activation and platelet aggregation [25]. Does their upregulation mean that patients using Roxadustat have increased cardiovascular risk? This is clearly an important area that needs further study.

### Validation by PRM

**Fig. 7:**
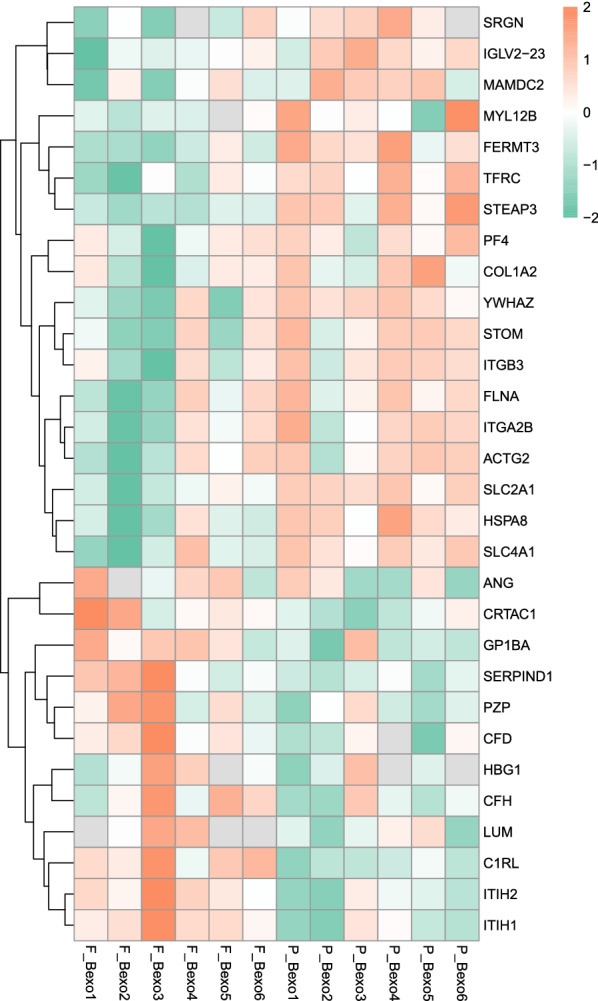
Heat map comparison of DEPs verified by PRM assay and label-free quantitative proteomics analysis

**Fig. 8:**
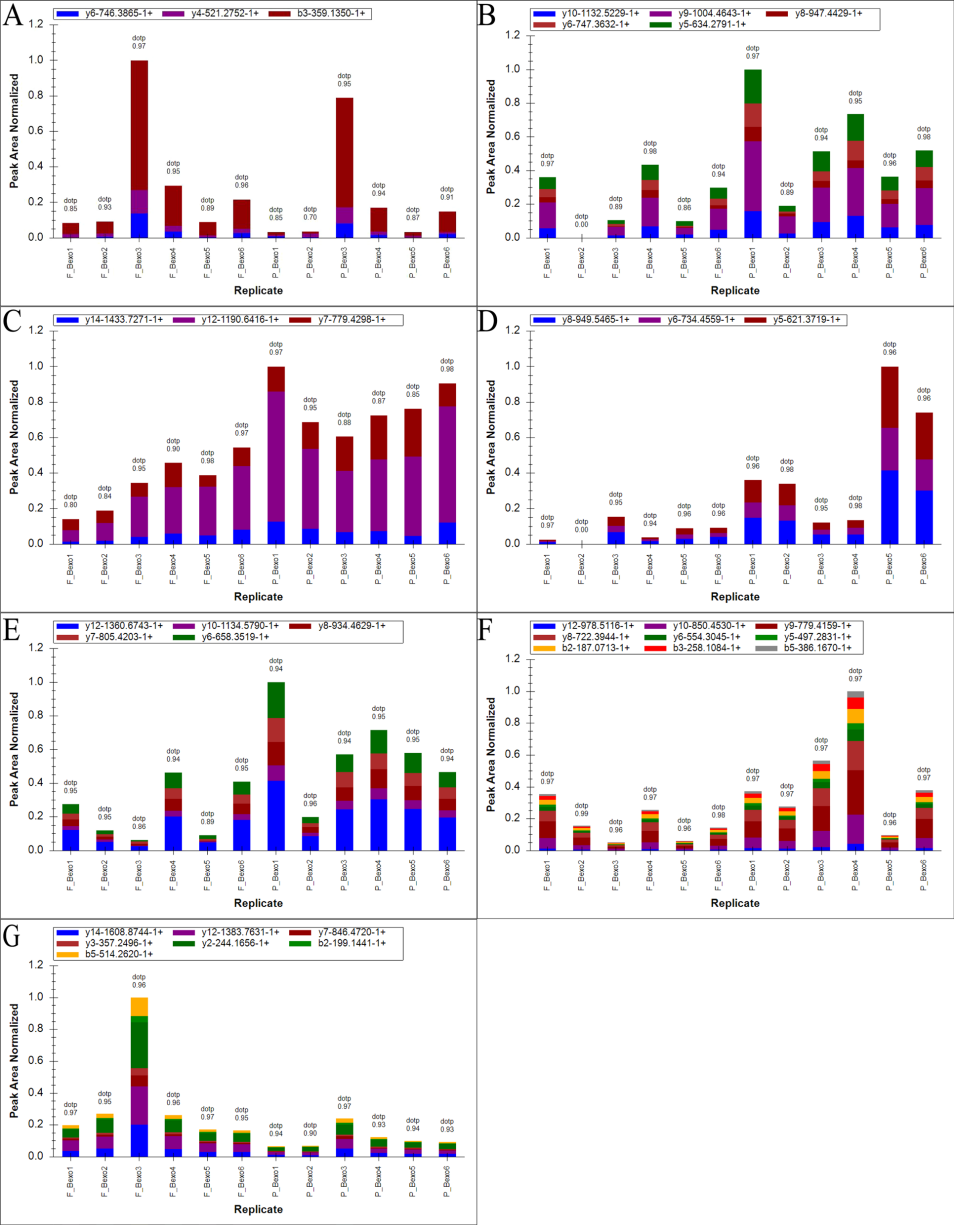
The fragment ion peak area distribution of identified peptides of differential proteins. **A** DGWSAQPTCIK (Protein P08603). **B** SVTEQGAELSNEER (Protein P63104). **C** NEPTAAAIAYGLDK (Protein P11142). **D** YNSQLLSFVR (Protein P02786). **E** DNCAPESIEFPVSEAR (Protein P05106). **F** GEAGAAGPAGPAGPR (Protein A0A087WTA8). **G** VNNSPQPQNVVFDVQIPK (Protein P19823)

The PRM assay was applied to validate the selected proteins obtained in the label-free quantitative proteomics analysis. Limited by the characteristics of some proteins and the abundance of their expression, we quantified 16 of them. As shown in Fig. 7, the changing trends of these 16 proteins in PRM analysis were consistent with the data from Label-free analysis (see Additional file 5), which further confirmed the credibility of the exosome data. However, 9 protein changes were not statistically significant, this is mainly because Data acquisition of label-free quantification (LFQ) was performed with the data-dependent acquisition (DDA) mode, which collects thousands of peptides and then compares with the database, and may have false-positive results sometimes. PRM is for the detection of specific proteins, and its sensitivity and accuracy are higher than LFQ. Therefore, the results of LFQ for some proteins may be inconsistent with the PRM results. Finally, the results are summarized and confirmed that TFRC, HSPA8, ITGB3, COL1A2, and YWHAZ were markedly upregulated, while ITIH2 and CFH were significantly downregulated upon treatment with roxadustat. The fragment ion peak of one peptide and corresponding proteins were shown in Fig. 8.

## Discussion

Roxadustat (FG-4592) is a HIF-PHI used to treat anemia in chronic kidney disease by stabilizing HIF. This study conducted LC-MS/MS label-free quantitative proteomics and PRM assay to compare protein profiles in patients' exosomes. Differential proteomic analysis shed light on 7 spots, all altered in abundance after one month of treatment: TFRC, HSPA8, ITGB3, COL1A2, YWHAZ, CFH, and ITIH2.

Negative iron balance is a critical contributor to anemia in CKD patients apart from inflammation and erythropoietin (EPO) deficiency [26]. Numerous studies have revealed that total iron-binding capacity increased considerably in patients receiving roxadustat, while serum hepcidin and ferritin levels were reduced in comparison to patients on placebo [27]. These changes in iron metabolism are not only regulated by the interaction of hepcidin and HIF [3], but also by other iron-related proteins, such as transferrin receptor(TFRC) [28]. TFRC is a type II transmembrane glycoprotein that is found on all nucleated cells, and the prominent function of TFRC is iron uptake by binding Fe3 + loaded transferrin and subsequent endocytosis [29], which is the part of Iron Regulatory Proteins-Iron-Responsive Elements(IRP–IRE) regulatory network, and the major source of iron for erythropoiesis is transferrin-bound iron uptake via TFRC [30]. Besides this, Lok et al. demonstrated that the TFRC gene contains a functional hypoxia response element (HRE) that binds HIF-1, which regulates receptor expression under hypoxic conditions [31]. In this experiment, we found that Roxadustat significantly improves the level of the TFRC (p = 0.00). Our results further confirmed that upregulated TFRC demonstrates that it may be an important target of Roxadustat to enhance erythropoiesis through different mechanisms.

ITI(Inter-alpha-trypsin inhibitor) family members have been demonstrated to be both positive and negative acute-phase proteins that are activated in the inflammation setting, such as pancreatitis [21], or sepsis [32]. As we know, inflammation also plays an important role in renal anemia [27]. According to estimations, more than 30%–50% of patients with end-stage renal disease (ESRD) have serological evidence of an active inflammatory state, such as elevated levels of CRP and pro-inflammatory cytokines. The expression level of Inter-alpha-trypsin inhibitor heavy chain H2(ITIH2) was markedly downregulated with Roxadustat treatment. Concordantly, we found its levels in patient 4 with the more severe condition were significantly higher than other patients before and after administration, although we did not find a positive correlation between ITIH level and relevant indicators such as serum creatinine or C-reactive protein due to the limited clinical data. Therefore, down-regulation of ITIH may illustrate that Roxadustat has an anti-inflammatory effect, and the ITIH family can be potential markers to reflect the severity of the inflammatory response and monitor the response of Roxadustat treatment.

Heat shock 70 kDa protein 8(HSPA8) is a member of HSP70 belonging to the heat shock protein family and plays an essential role in many biological processes [33]. Studies indicated that HSPA8 could enhance the stability of BCL2L11/BIM mRNA stability to regulate the total counts of hematopoietic cells with the help of HSP40 proteins, HIP, BAG4, and STUB1 [34]. Meantime, Jinfen et al. compared the differences of the glycolytic score and key glycolytic gene expression at different hypoxia states and HSPA8 expression patterns, the data show that HSPA8 is strongly associated with glycolysis activity and correlated with key glycolytic genes such as PGK1. In the present study, HSPA8 was significantly upregulated after Roxadustat treatment (P = 0.00). Based on this evidence, we speculate that HSPA8 may be one of active sites of Roxadustat to act on hematopoietic stem cells to improve the anemia, and may affect glycolysis by regulation of glycolytic genes in hypoxia environment by up-regulating the expression of HSPA8. In 2020, the Japanese scholar Hiroko Deguchi et al. reported that preconditioning with Roxadustat can reduce myocardial ischemic injury by a respiratory metabolic shift [35]. This might be because increased glycolysis can maintain myocytes under ATP-starved conditions, conferring cardio protection against ischemic injury. Therefore, our study provides a theoretical basis for the application potential of Roxadustat for reducing ischemic injury.

Does Roxadustat affect platelet production and function? Some authors suggest that Roxadustat does not cause platelet activation, but the KEGG-based functional enrichment analysis showed that many DEPs are involved in the signal pathway of platelet activation, such as ITGA2B、ITGB3、COL1A1、COL1A2. It is well known that CKD patients are at a higher risk of thrombosis than people with normal kidney function [36], and the use of ESAs in patients with renal anemia can increase the risk of hypercoagulable [37]. Our results could call into question the hypothesis that Roxadustat does not induce platelet activation, and multicenter prospective randomized clinical trials with a larger sample size are needed to further verify our results.

## Conclusions

Overall*,* this study used the label-free LC-MS/MS technology platform to screen out the differential proteins in plasma exosomes related to Roxadustat treatment. Our results stated that TFRC and HSPA8 could be an important target of the action of Roxadustat, and Roxadustat may increase cardiovascular risk through its influence on platelet activation. Of course, there are several limitations to our work. First, this study is a self-controlled study before and after Roxadustat treatment. There is a lack of placebo as a randomized control. Second, our study was limited by sample size. Third, these evaluations are only preliminary and should be confirmed with deeper basic research and clinical practice. Future work will address these questions.

## Supplementary Information

**Additional file 1: **List of DEPs in exosomes between CKD patients.

**Additional file 2: **Pathway analysis of differential genes: hsa04611 Platelet activation. Red marks indicate the genes with differential profiles.

**Additional file 3: **Pathway analysis of differential genes: hsa04066 HIF-1 signaling pathway. Red marks indicate the genes with differential profiles.

**Additional file 4: **Pathway analysis of differential genes: hsa04216 Ferroptosis. Red marks indicate the genes with differential profiles.

**Additional file 5: **Basic information of differentially expressed proteins verified by PRM.

## Data Availability

All data generated or analyzed during this study are included in this published article and its supplementary information files.

## Abbreviations

CKD: Chronic kidney disease
HIF-PHI: Hypoxia-inducible factor prolyl hydroxylase inhibitor
EPO: Erythropoietin
DEPs: Differentially expressed proteins
GO: Gene ontology
KEGG: Kyoto encyclopedia of genes and genomes
PPI: Protein–protein interaction
KDOQI: Kidney disease outcomes quality initiative
BPS: Blood pureexo solution
TEM: Transmission electron microscopy
NTA: Nanoparticle tracking analysis
TEAB: Triethylammonium bicarbonate
TFA: Trifluoroacetic acid
DDA: Data dependant scan
AGC: Data dependant scan
FDR: False discover rate
PRM: Parallel reaction monitoring
FDR: False discovery rate
SEM: Standard error of the mean
HB: Hemoglobin
PSM: Peptide-spectrum matches
TSH: Thyroid-stimulating hormone
TFRC: Transferrin receptor
IRP–IRE: Iron regulatory proteins-Iron-responsive elements
HRE: Hypoxia response element
ITI: Inter-alpha-trypsin inhibitor
ESRD: End-stage renal disease
ITIH2: Inter-alpha-trypsin inhibitor heavy chain H2
HSPA8: Heat shock 70 kDa protein 8
