# Supplementary figures and images for: Label-free quantitative proteomic analysis of serum exosomes from patients of renal anemia: The Good and the Bad of Roxadustat
Source: Clin Proteomics. 2022 Jun 11;19:21. doi: 10.1186/s12014-022-09358-w (PMC9187900; doi:10.1186/s12014-022-09358-w)

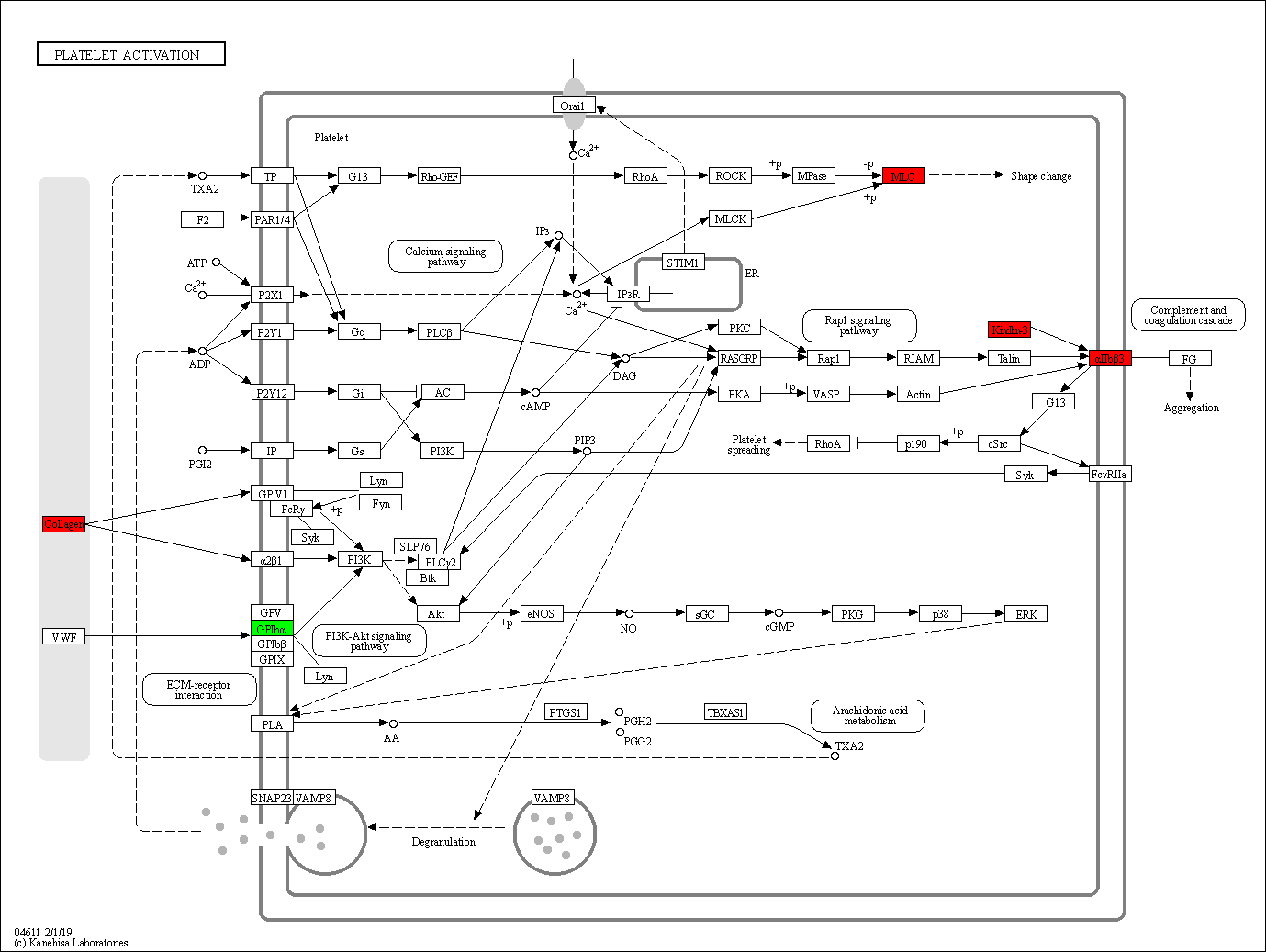

Supplement: Supplementary file 2 — Additional file 2: Pathway analysis of differential genes: hsa04611 Platelet activation. Red marks indicate the genes with differential profiles. [file 12014_2022_9358_MOESM2_ESM.png]

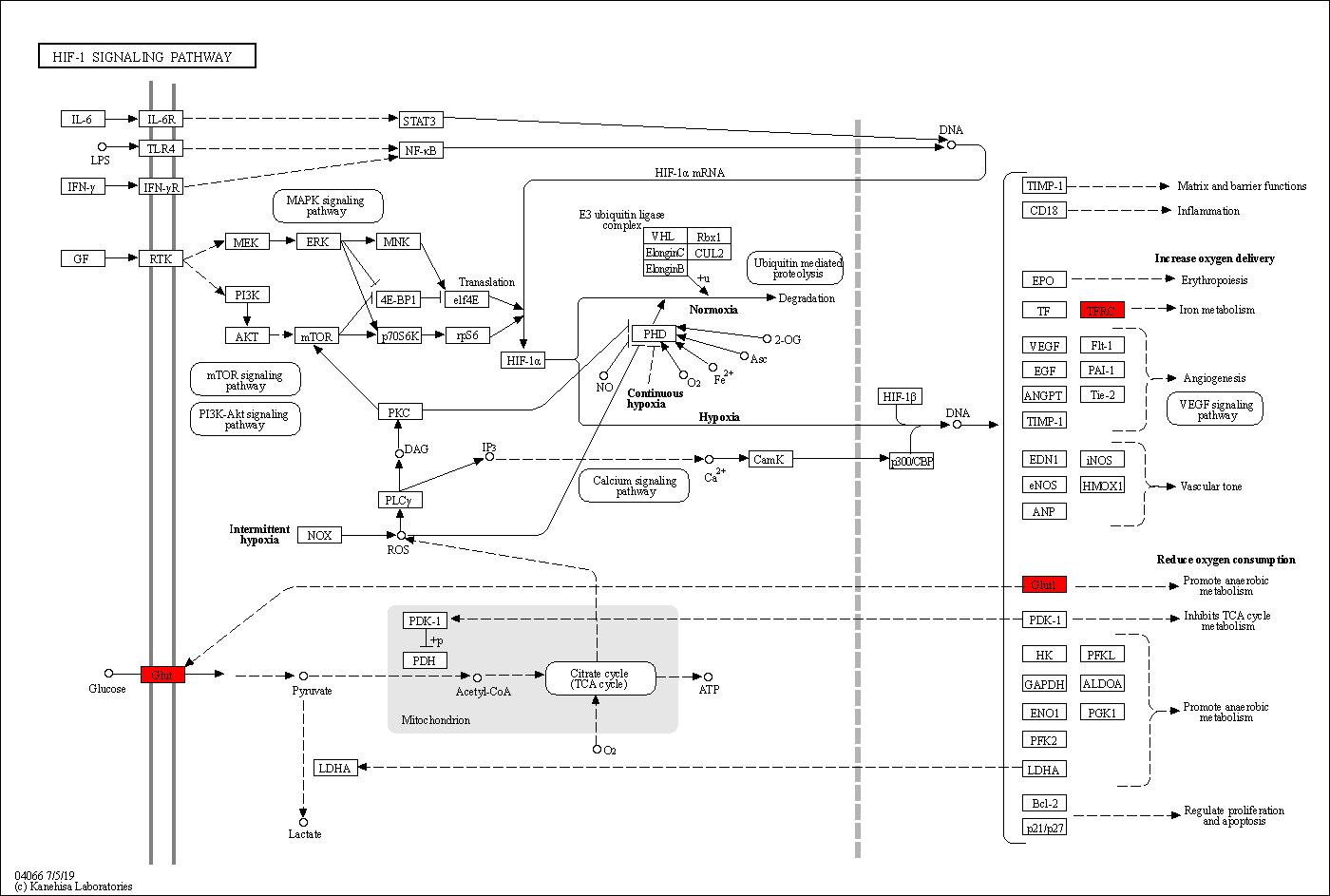

Supplement: Supplementary file 3 — Additional file 3: Pathway analysis of differential genes: hsa04066 HIF-1 signaling pathway. Red marks indicate the genes with differential profiles. [file 12014_2022_9358_MOESM3_ESM.png]

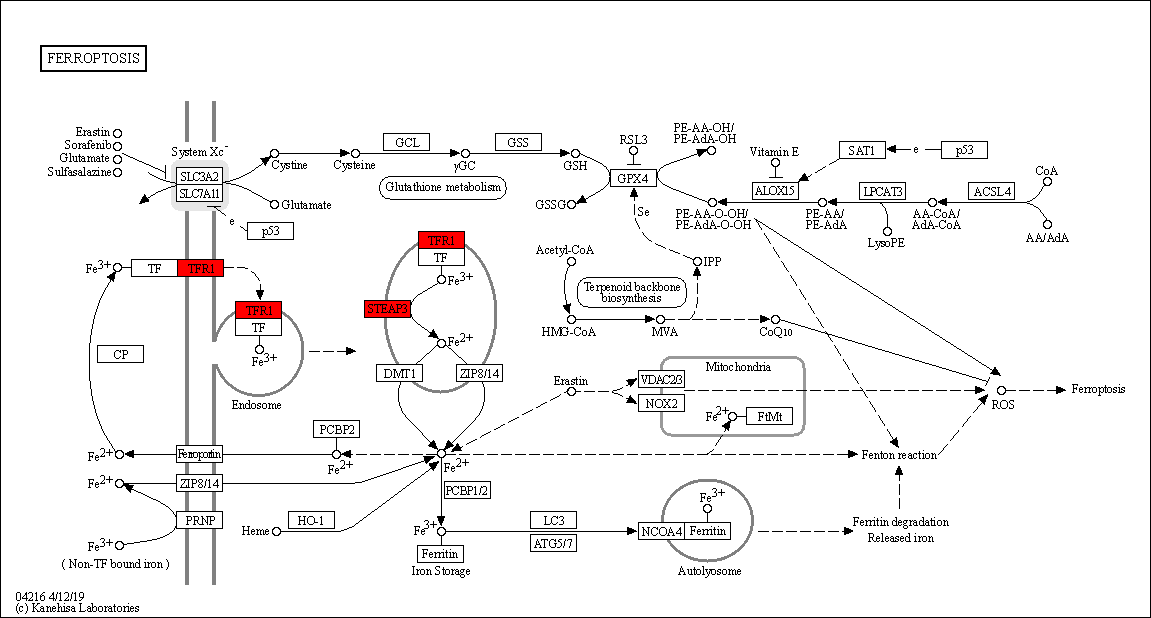

Supplement: Supplementary file 4 — Additional file 4: Pathway analysis of differential genes: hsa04216 Ferroptosis. Red marks indicate the genes with differential profiles. [file 12014_2022_9358_MOESM4_ESM.png]
